# Efficacy and Safety of Direct Oral Anticoagulants in Patients with Diabetes and Nonvalvular Atrial Fibrillation: Meta-Analysis of Observational Studies

**DOI:** 10.1155/2021/5520027

**Published:** 2021-10-11

**Authors:** Bo Cao, Xingcan Yao, Lifang Zhang, Xiaobo Hu, Min Chen, Mingfeng Shen, Lan Xu

**Affiliations:** ^1^Department of Clinical Pharmacy, Affiliated Ninth Hospital of Suzhou University, Suzhou, China; ^2^Department of Cardiovascular Medicine, Affiliated Ninth Hospital of Suzhou University, Suzhou, China

## Abstract

**Background:**

This meta-analysis was performed to compare the efficacy and safety of direct oral anticoagulants (DOACs) with vitamin K antagonists (VKAs) for stroke prevention in real-world patients with diabetes and nonvalvular atrial fibrillation (NVAF) through observational studies.

**Methods:**

PubMed, Embase, and Web of Science databases were searched up to August 2020 for eligible studies. Outputs were presented as risk ratios (RRs) and corresponding 95% confidence intervals (CIs) by using a random-effect model.

**Results:**

Seven observational studies involving 249,794 diabetic NVAF patients were selected. Compared with VKAs, the use of DOACs was associated with significantly reduced risks of stroke (RR = 0.56, 95% CI 0.45-0.70; *p* < 0.00001), ischemic stroke (RR = 0.61, 95% CI 0.48-0.78; *p* < 0.0001), stroke or systemic embolism (SSE) (RR = 0.81, 95% CI 0.68-0.95; *p* = 0.01), myocardial infarction (RR = 0.69, 95% CI 0.55-0.88; *p* = 0.002), major bleeding (RR = 0.75, 95% CI 0.63-0.90; *p* = 0.002), intracranial hemorrhage (RR = 0.50, 95% CI 0.44-0.56; *p* < 0.00001), and major gastrointestinal bleeding (RR = 0.77, 95% CI 0.62-0.95; *p* = 0.02), and a borderline significant decrease in major adverse cardiac events (RR = 0.87, 95% CI 0.75-1.00; *p* = 0.05) in NVAF patients with diabetes.

**Conclusion:**

For patients with NVAF and diabetes in real-world clinical settings, DOACs showed superior efficacy and safety profile over VKAs and significantly reduced risks of stroke, ischemic stroke, SSE, myocardial infarction, major bleeding, intracranial hemorrhage, and major gastrointestinal bleeding.

## 1. Introduction

Atrial fibrillation (AF) is the most common cardiac arrhythmia and an independent risk factor for stroke [1]. Diabetes mellitus (DM) is a common comorbidity in AF patients, and the prevalence of AF is at least twofold higher in patients with DM than in those without DM [2]. DM increases the incidence of major adverse cardiac events (MACE), such as stroke, myocardial infarction, and cardiovascular death, in patients with AF compared with those without AF [3]. Accordingly, DM has been an independent risk factor for the prediction of stroke in CHA_2_DS_2_-VASc [4]. Therefore, diabetic AF patients are a high-risk subgroup; prophylactic oral anticoagulation is crucial for this population to reduce the excessive risk of cardiovascular events [5, 6].

Although traditional vitamin K antagonists (VKAs) have great efficacy in AF patients [7], the required monitoring of the international normalized ratio (INR), frequent dose adjustment, and interaction with other drugs or food make this treatment inconvenient and burdensome [8–10]. Hence, direct oral anticoagulants (DOACs) have been developed and introduced to be an innovation for preventing thromboembolic complications over the past decade. The four DOACs, i.e., apixaban, dabigatran, edoxaban, and rivaroxaban, showed noninferior efficacy and safety profiles compared with warfarin in randomized controlled trials [11].

A meta-analysis of the four DOAC randomized controlled trials showed that DOACs had similar efficacy and safety profiles to warfarin in patients with diabetes and nonvalvular AF (NVAF) [12]. However, only a few observational studies evaluated and compared the real-world efficacy and safety of DOACs and VKAs in diabetic NVAF patients. Moreover, the effect of DOACs on MACE is seldom evaluated compared with that of VKAs in patients with NVAF and DM. On the basis of recently updated real-world comparison studies of DOACs with VKAs, a meta-analysis was conducted to systematically evaluate the clinical outcomes of DOACs in patients with NVAF and DM and compare the efficacy and safety of DOACs versus VKAs in a real-world setting.

## 2. Methods

The analysis was established according to the Meta-analysis Of Observational Studies in Epidemiology (MOOSE) [13].

### 2.1. Literature Search

PubMed, Embase, and Web of Science were systematically searched until August 2020 for relevant studies comparing the effect between DOAC and VKA in patients with AF and diabetes. The detailed search strategy was as follows: (1) atrial fibrillation OR AF OR nonvalvular atrial fibrillation AND (2) diabetes AND (3) non-vitamin K antagonist oral anticoagulants OR NOACs OR direct oral anticoagulants OR DOACs OR new oral anticoagulants OR novel oral anticoagulants OR oral thrombin inhibitors OR factor Xa inhibitors OR dabigatran OR rivaroxaban OR apixaban OR edoxaban; AND (4) vitamin K antagonists OR warfarin. For a comprehensive search, the reference lists of retrieved studies were handsearched to identify additional reports. No linguistic restrictions were applied.

### 2.2. Eligibility Criteria

Eligibility criteria were as follows: (1) observational studies such as prospective or retrospective cohorts; (2) studies comparing the outcomes of any DOAC (dabigatran, rivaroxaban, apixaban, or edoxaban) and warfarin in AF patients with diabetes, such as stroke or systemic embolism (SSE), ischemic stroke (IS), myocardial infarction (MI), major adverse cardiac events (MACE), major bleeding, intracranial hemorrhage (ICH), and gastrointestinal (GI) bleeding in patients with AF and diabetes; (3) studies published in peer-reviewed journals with full text available; and (4) the study with the longest period or the largest sample size was included when the subjects across studies were from the same data source. Articles matching clinical trials, exclusive cardioversion or catheter ablation studies, case reports, reviews, editorials, letters, animal studies, and publications with no data were excluded.

### 2.3. Data Extraction and Study Quality Assessment

The retrieved literature found during the database search was screened by two authors (B Cao and XC Yao) independently. The studies were included according to the inclusion criteria after abstract reading or full-text review. The final selection of studies was performed by consensus or discussion with a third author (XB Hu). Study characteristics including the following data were documented: the first author and publication year, study design, inclusion period, demographic and clinical characteristics of the patients, type of DOACs, sample size, and follow-up duration.

Study quality was evaluated according to the modified Newcastle-Ottawa Scale (NOS) tool, which includes three domains: selection (0–4 points), comparability (0–2 points), and exposure (0–3 points). Specific information is presented in Supplemental Table 1. A study with an NOS score ≥ 6 was defined as having moderate-to-high quality [14].

### 2.4. Statistical Analysis

All of the statistical analyses were performed using the Review Manager 5.3 software (the Nordic Cochrane Center, Rigshospitalet, Denmark) and the Stata software (version 14.0, Stata Corp. LP, College Station, TX). We collected the number of events and sample size of each cohort. The expected number of events was calculated based on event rates if the number of events was not available: event number = (total patient number) × [event rate (per 100 patient − years)] × [follow − up time (years)] [15]. The risk ratio (RR) with 95% confidence interval (CI) was calculated for each included study, and then pooled by a random-effect model using the Mantel-Haenszel method. The Cochrane *Q* test and *I*^2^ statistic were the most commonly used statistical methods to evaluate heterogeneity, where *p* < 0.1 and *I*^2^ > 50% indicated a substantial heterogeneity. The method of exclusion of one study at a time was used for sensitivity analysis. The publication bias was assessed using the funnel plots and further calculated using the Egger tests. Subgroup analysis was also performed based on the type of NOAC (apixaban, dabigatran, rivaroxaban, or edoxaban). *p* < 0.05 was considered statistically significant.

## 3. Results

### 3.1. Study Selection

A total of 495 articles were identified through the systemic database search. After the duplicates and studies that did not meet the eligibility criteria were excluded, seven studies [16–22] were included (Figure S1). The baseline characteristics of selected studies are shown in Supplementary Table S2. All studies were retrospective and included 249,794 patients, 130,760 of which were treated with DOACs and the remaining 119,034 with VKAs. Definitions of safety and efficacy endpoints in the seven included studies are presented in Supplementary Table S3. All included studies had acceptable quality with an NOS score of ≥6 (Supplementary Table S1).

### 3.2. Efficacy Outcomes of DOAC versus VKA

**Figure 1:**
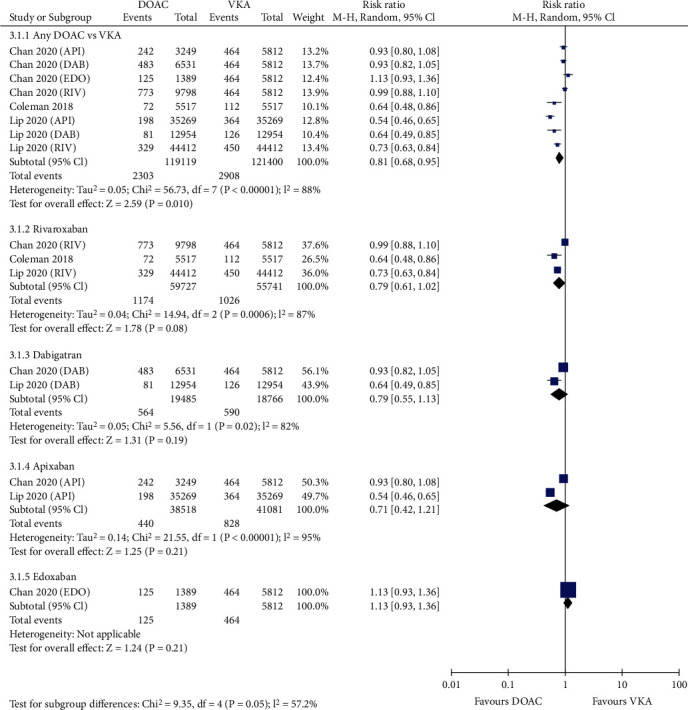
Forest plot comparing DOACs vs. VKAs regarding SSE in real-world NVAF patients with diabetes. SSE: stroke or systemic embolism; NVAF: nonvalvular atrial fibrillation; DOACs: direct oral anticoagulants; VKAs: vitamin K antagonists; API: apixaban; DAB: dabigatran; EDO: edoxaban; RIV: rivaroxaban.

**Figure 2:**
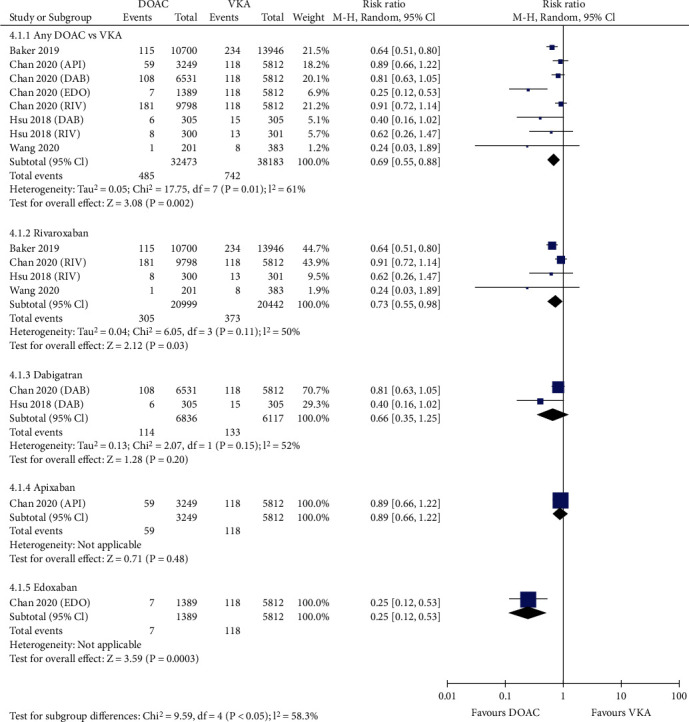
Forest plot comparing DOACs vs. VKAs regarding myocardial infarction in real-world NVAF patients with diabetes. NVAF: nonvalvular atrial fibrillation; DOACs: direct oral anticoagulants; VKAs: vitamin K antagonists; API: apixaban; DAB: dabigatran; EDO: edoxaban; RIV: rivaroxaban.

**Figure 3:**
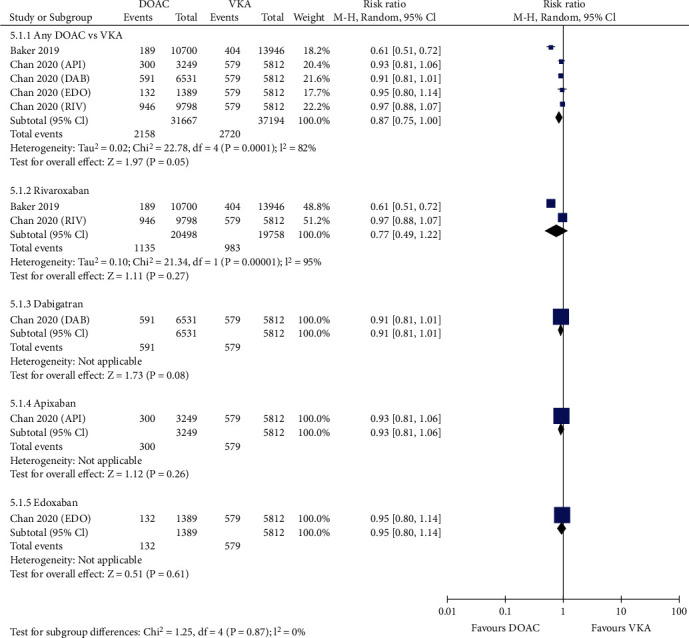
Forest plot comparing DOACs vs. VKAs regarding MACE in real-world NVAF patients with diabetes. MACE: major adverse cardiac events; NVAF: nonvalvular atrial fibrillation; DOACs: direct oral anticoagulants; VKAs: vitamin K antagonists; API: apixaban; DAB: dabigatran; EDO: edoxaban; RIV: rivaroxaban.

Figure S2 and Figure S3 shows that compared with VKAs, the use of DOACs was associated with significantly lower risks of stroke (0.66% vs. 1.12%, RR = 0.56, 95% CI 0.45-0.70; *p* < 0.00001; Figure S2) and ischemic stroke (0.58% vs. 0.91%, RR = 0.61, 95% CI 0.48-0.78; *p* < 0.0001; Figure S3). DOACs also considerably reduced the risk of stroke or systemic embolism (1.93% vs. 2.40% in the VKA group, RR = 0.81, 95% CI 0.68-0.95; *p* = 0.01; Figure 1). In four studies reporting myocardial infarction, the risk was significantly reduced in patients treated with DOACs compared with those treated with VKAs (1.49% vs. 1.94%, RR = 0.69, 95% CI 0.55-0.88; *p* = 0.002; Figure 2). However, the use of DOACs borderline significantly reduced the rate of MACE compared with VKAs (6.81% vs. 7.31%, RR = 0.87, 95% CI 0.75-1.00; *p* = 0.05; Figure 3).

Stratified analyses regarding efficacy were also conducted according to the anticoagulant mechanism of DOACs (Table 1). Compared with VKAs, anti-IIa agents (dabigatran) and anti-Xa agents (apixaban, edoxaban, and rivaroxaban) significantly reduced the risk of stroke (anti-IIa agents: RR = 0.64; 95% CI 0.48-0.85; *p* = 0.002; anti-Xa agents: RR = 0.54; 95% CI 0.41-0.71; *p* < 0.0001). With regard to MACE, the two types of DOAC agents showed similar rates versus VKAs (anti-IIa agents: RR = 0.91; 95% CI 0.81-1.01; *p* = 0.084; anti-Xa agents: RR = 0.85; 95% CI 0.70-1.04; *p* = 0.112). However, anti-Xa agents were associated with significantly decreased risks in ischemic stroke (RR = 0.58; 95% CI 0.43-0.77; *p* < 0.0001), myocardial infarction (RR = 0.67; 95% CI 0.50-0.91; *p* = 0.011), and SSE (RR = 0.81; 95% CI 0.65-1.00; *p* = 0.047) compared with VKAs. However, no difference was observed for anti-IIa agents versus VKAs (ischemic stroke: RR = 0.80; 95% CI 0.58-1.10; *p* = 0.17; myocardial infarction: RR = 0.66; 95% CI 0.35-1.25; *p* = 0.201; SSE: RR = 0.79; 95% CI 0.55-1.13; *p* = 0.19). This finding indicated the favorable efficacy profile of anti-Xa agents over anti-IIa agents.

### 3.3. Safety Outcomes of DOAC versus VKA

**Figure 4:**
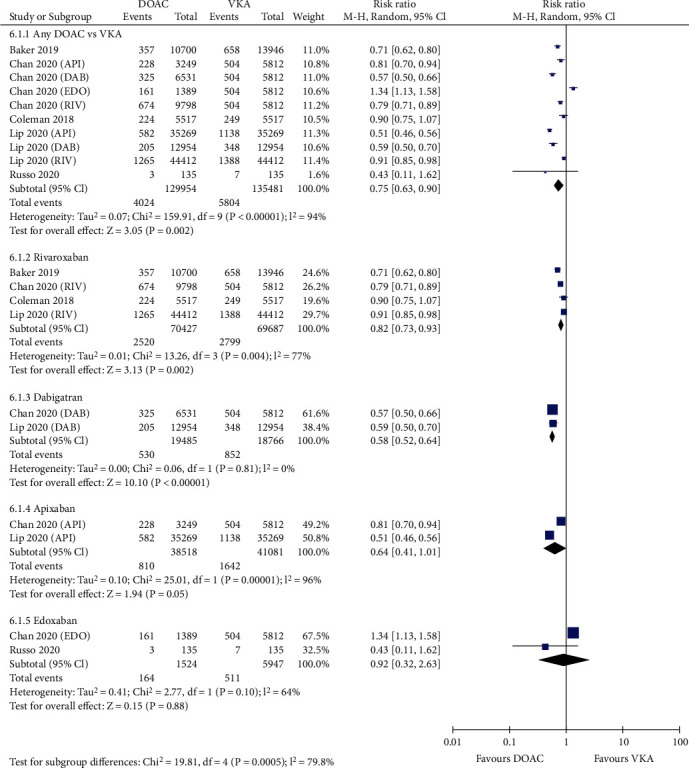
Forest plot comparing DOACs vs. VKAs regarding major bleeding in real-world NVAF patients with diabetes. NVAF: nonvalvular atrial fibrillation; DOACs: direct oral anticoagulants; VKAs: vitamin K antagonists; API: apixaban; DAB: dabigatran; EDO: edoxaban; RIV: rivaroxaban.

**Figure 5:**
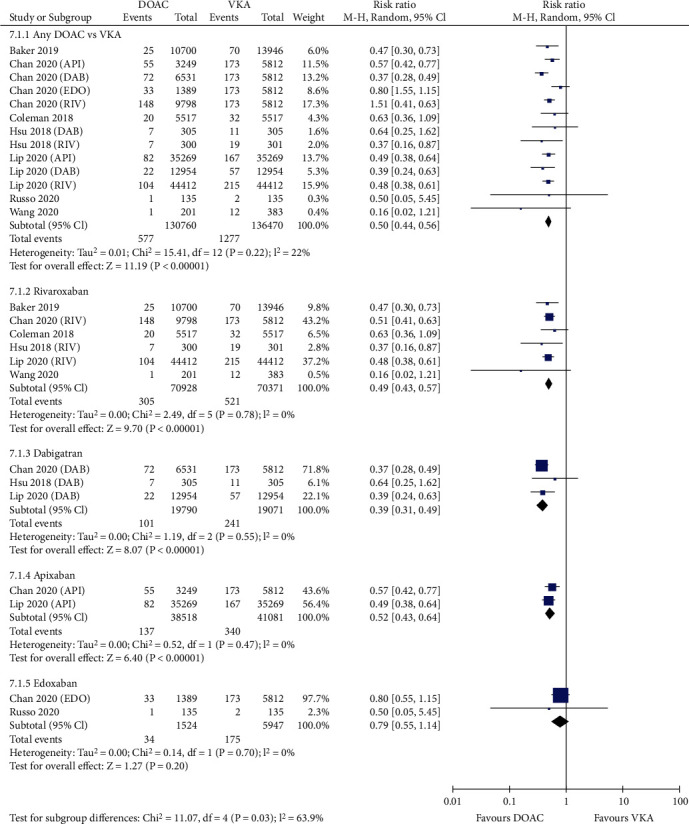
Forest plot comparing DOACs vs. VKAs regarding intracranial hemorrhage in real-world NVAF patients with diabetes. NVAF: nonvalvular atrial fibrillation; DOACs: direct oral anticoagulants; VKAs: vitamin K antagonists; API: apixaban; DAB: dabigatran; EDO: edoxaban; RIV: rivaroxaban.

**Figure 6:**
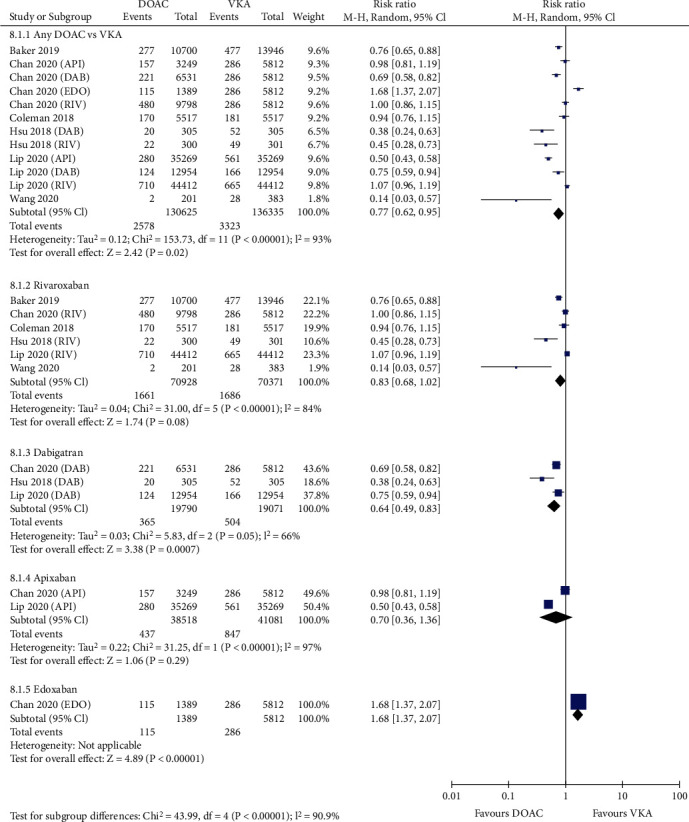
Forest plot comparing DOACs vs. VKAs regarding major gastrointestinal bleeding in real-world NVAF patients with diabetes. NVAF: nonvalvular atrial fibrillation; DOACs: direct oral anticoagulants; VKAs: vitamin K antagonists; API: apixaban; DAB: dabigatran; EDO: edoxaban; RIV: rivaroxaban.

Figure 4 shows that compared with VKAs, the use of DOACs was associated with a decreased risk of major bleeding (3.10% vs. 4.28%, RR = 0.75, 95% CI 0.63-0.90; *p* = 0.002). In the seven studies reporting intracranial hemorrhage, DOACs showed a significantly reduced incidence rate compared with VKAs (0.44% vs. 0.94%, RR = 0.50, 95% CI 0.44-0.56; *p* < 0.00001; Figure 5). For the six studies reporting major gastrointestinal bleeding, the risk was considerably lower in patients treated with DOACs than in those treated with VKAs (1.97% vs. 2.44%, RR = 0.77, 95% CI 0.62-0.95; *p* = 0.02; Figure 6).

Stratified analyses based on the anticoagulant mechanism of DOACs regarding safety were also performed (Table 2). As shown in Table 2, the anti-IIa agents significantly decreased the incidence of major bleeding (RR = 0.58; 95% CI 0.52-0.64; *p* < 0.0001) and intracranial hemorrhage (RR = 0.39; 95% CI 0.31-0.49; *p* < 0.0001) compared with VKAs, as well as the anti-Xa agents (major bleeding: RR = 0.81; 95% CI 0.66-0.99; *p* = 0.044; intracranial hemorrhage: RR = 0.52; 95% CI 0.47-0.59; *p* < 0.0001). However, in terms of major gastrointestinal bleeding, the use of anti-IIa agents was associated with significantly reduced risks rather than comparable rates of anti-Xa agents versus VKAs (anti-IIa agents: RR = 0.64; 95% CI 0.49-0.83; *p* = 0.001; anti-Xa agents: RR = 0.83; 95% CI 0.64-1.07; *p* = 0.15). This finding indicated better safety performance of anti-IIa agents than anti-Xa agents.

### 3.4. Sensitivity and Subgroup Analysis

Sensitivity analysis was performed by excluding one study at a time. If the pooled effect did not change substantially, then the results are reliable. As shown in Figures 1–6, the subgroup analysis was performed based on the DOAC type (dabigatran, rivaroxaban, apixaban, and edoxaban). Compared with VKAs, dabigatran, rivaroxaban, apixaban, and edoxaban had lower or similar rates of thromboembolic events (Figures 1–3). All individual DOACs showed similar or superior safety profiles regrading major bleeding and intracranial hemorrhage (Figures 4–5). However, the risk of gastrointestinal bleeding was significantly higher in edoxaban than in VKA (RR = 1.68, 95% CI 1.37-2.07; *p* < 0.00001; Figure 6), and this result differed from those for the three other DOAC types.

### 3.5. Publication Bias

For the meta-analysis of the pooled effect regarding efficacy and safety outcomes, publication bias was determined as inspected by the funnel plots (Figure S4). However, Egger's test results for one outcome indicated certain publication bias (Figure S5). Therefore, trim-and-fill analysis was conducted to adjust for funnel plot asymmetry. The results showed no trimming and unchanged results.

## 4. Discussion

In this meta-analysis of seven real-world observational studies with 249,794 patients with NVAF and diabetes, the use of DOACs was associated with significantly lower risks of stroke, ischemic stroke, SSE, myocardial infarction, major bleeding, intracranial hemorrhage, and major gastrointestinal bleeding and a borderline significantly reduced rate of MACE compared with VKAs. Moreover, individual DOACs versus VKAs showed similar or reduced rates of thromboembolic and bleeding events except for edoxaban in gastrointestinal bleeding.

Stratified analyses based on anticoagulant mechanism revealed that anti-Xa agents (apixaban, edoxaban, and rivaroxaban) and anti-IIa agents (dabigatran) showed similar results in reducing the incidence of stroke, major bleeding, and intracranial hemorrhage compared with VKAs. However, anti-Xa agents significantly reduced the risks of ischemic stroke, myocardial infarction, and SSE compared to VKAs than anti-IIa agents. This finding indicated the more favorable efficacy profile of anti-Xa agents over dabigatran. Conversely, compared with VKAs, dabigatran decreased significantly lower risks of gastrointestinal bleeding than anti-Xa agents. This result showed the superior safety profile of dabigatran over apixaban, edoxaban, and rivaroxaban. The efficacy and safety of individual DOACs against each other have been reported. One meta-analysis on randomized controlled trials [23] indirectly compared the efficacy and safety of dabigatran, apixaban, and rivaroxaban and showed that apixaban was associated with less major bleeding than dabigatran 150 mg or rivaroxaban and that rivaroxaban was less effective than dabigatran 150 mg in preventing stroke or systemic embolism. In another retrospective cohort study based on Asian patients with NVAF [24], rivaroxaban induced a significantly higher risk for gastrointestinal bleeding than dabigatran. A new-user cohort study on elderly patients with NVAF evaluated each individual DOAC [25] and reported that dabigatran and apixaban were associated with more favorable benefit-harm profile than rivaroxaban. Another meta-analysis of real-world studies in patients with NVAF [26] found that rivaroxaban was associated with significantly higher risk of major bleeding and gastrointestinal bleeding than dabigatran. Despite the varying efficacy outcomes of each DOAC across studies [23–26], the present stratified analyses of safety outcomes consisted of studies showing that dabigatran had a better safety profile than the three other DOACs in patients with NVAF and diabetes.

To the best of the authors' knowledge, this meta-analysis of real-world studies is the first to investigate the efficacy and safety of DOACs versus VKAs in NVAF patients with DM and included recently updated observational studies especially regarding edoxaban. The four landmark DOAC trials included certain proportions of NVAF patients with DM, 39.9% in the ROCKET AF trial with rivaroxaban [27], 23.3% in the RE-LY trial with dabigatran [28], 25% in the ARISTOTLE trial with apixaban [29], and 36% in the ENGAGE AF-TIMI 48 trial with edoxaban [30]. In the post hoc analysis of the ROCKET AF study [31], rivaroxaban showed comparable risks of SSE and major bleeding to warfarin in NVAF patients regardless of diabetic status. The present subgroup results showed similar efficacy but better safety outcomes of rivaroxaban versus VKAs in diabetic NVAF patients. The post hoc analysis of the RE-LY trial [32] showed a comparable risk of major bleeding in NVAF patients with DM treated with dabigatran or warfarin. This finding was in contrast to our results with a significantly lower risk of dabigatran compared with VKAs. In the post hoc analysis of the ARISTOTLE trial [33], apixaban was the same as warfarin in the case of major bleeding among patients with diabetes and NVAF, and the present subgroup analysis was consistent with this finding. Finally, the post hoc analysis of the ENGAGE AF-TIMI 48 study [34] indicated that edoxaban reduced more major bleeding than warfarin both in the diabetic and nondiabetic groups, while our results showed a comparable safety outcome for edoxaban versus VKAs regarding major bleeding in patients with NVAF and DM.

The meta-analysis of the four DOAC trials revealed that DOACs significantly reduced the risks of stroke/SE and major bleeding compared with warfarin in NVAF patients with or without diabetes [12], suggesting that diabetic status has no differential effect on efficacy or safety endpoints. Another review [35] of the four DOAC trials with post hoc analyses showed similar results that DOACs are safe and can reduce the incidence of major bleeding. This result suggested that the efficacy and safety of DOACs over VKAs generally extend to NVAF patients with DM. A new meta-analysis of the four DOAC trials [36] extended the results with added breadth and depth of the data and showed that DOACs reduced stroke/SE by 20%, intracranial hemorrhage by 49%, and total mortality by 10% compared with warfarin in diabetic NVAF patients. No significant differences in the magnitude of reduction was observed between the specific DOACs.

Patients in the DOAC trials do not always represent those in real-world settings. Therefore, information from observational studies of patients in daily practice must be obtained. Only a few retrospective studies evaluated the clinical outcomes of DOACs in NVAF patients with DM, and one meta-analysis investigated the efficacy and safety of rivaroxaban in this population [37]. The results revealed that rivaroxaban was associated with lower risks of stroke, ischemic stroke, SSE, major bleeding, and intracranial hemorrhage compared with warfarin, indicating the better efficacy and safety profile of the former. With recently updated observational studies (in particular edoxaban [20, 22]), the present meta-analysis evaluated the efficacy and safety of four DOACs in patients with NVAF and diabetes. Consistent with previous studies [12, 37], the present results showed that DOACs significantly reduced risks of stroke, ischemic stroke, SSE, and myocardial infarction compared with VKAs, suggesting its efficacy over VKAs in patients with diabetes and NVAF. The rates of major bleeding, intracranial hemorrhage, and major gastrointestinal bleeding were also much lower in patients prescribed with DOACs than those treated with VKAs. This finding indicated the safety of DOACs over VKAs in NVAF patients with diabetes. In summary, this work revealed the advantage of DOACs over VKAs regarding efficacy and safety in patients with NVAF and diabetes.

In six of the included studies [16–18, 20–22], the standard dose and reduced dose of DOACs were prescribed for patients with NVAF and diabetes. However, only three studies [16, 17, 21] evaluated the efficacy and safety outcomes by subgroup analysis based on dosage. Therefore, subgroup analysis based on DOAC dose could not be performed due to limited data. In the study of Lip et al. [21], the ratio of patients treated with a lower dose was 25.2% in apixaban (2.5 mg qd), 19% in dabigatran (75 mg bid), and 32% in rivaroxaban (15 or 10 mg qd). Subgroup analysis stratified by dosage indicated similar results for each DOCA in standard and reduced-dose groups. In Coleman et al.'s research [17], 20% of patients were prescribed with a low dose (rivaroxaban, 15 mg qd), but only the reduced-dose group showed significantly decreased risks of SSE and ischemic stroke. Any-dose and standard-dose analyses revealed similar efficacy and safety with warfarin. On the contrary, 24.1% of reduced-dose patients (rivaroxaban, 15 mg qd) in Baker et al.'s study [16] showed comparable effect on MACE versus warfarin use; however, the all-dose analysis indicated a significant protective effect. The number of patients receiving a reduced dose in three other studies were 88.5% of dabigatran (110 mg bid) and 87.5% of rivaroxaban (15 mg qd) in Hsu et al.'s study [18]; 66% of apixaban (2.5 mg qd), 89% of dabigatran (110 mg bid), 68% of edoxaban (30 mg qd), and 95% of rivaroxaban (15 or 10 mg qd) in Chan et al.'s research [20]; and 13% of edoxaban (30 mg qd) in Russo et al.'s study [22]. A tremendously higher prevalence of reduced-dose DOAC prescriptions was found in Asian patients with diabetes and AF [18, 20] than in the non-Asian population [16, 17, 21, 22] in the included studies. Asian patients with NVAF have higher risks of stroke and bleeding (in particular intracranial bleeding) than non-Asians [38–41]. Therefore, low-dose DOAC prescription is highly favorable for Asian patients.

Diabetes mellitus is related to a high risk of AF and poor recovery outcomes. Patients with diabetes had a 35% higher risk of AF than those without diabetes [5], and individuals with NVAF and diabetes had a 1.7-fold increased risk of stroke and worse prognosis [42, 43]. In addition, patients with diabetes who experienced stroke mostly had higher rates of mortality than those without diabetes [3, 44]. This phenomenon may be explained by the hypercoagulability state of this population. Mechanisms involved in this case include increased tissue plasminogen activator antigen, improved factor VIII activity, reduced fibrinolytic activity, and platelet and endothelial dysfunction [45, 46]. Therefore, the high-risk features of diabetic NVAF patients must be considered when developing the efficacy and safety of anticoagulation strategies in patient-specific management.

Persistence of anticoagulant treatment is important in the management of anticoagulation among diabetic NVAF patients, and polypharmacy usually occurs in this population and affects clinical outcomes [47]. However, good adherence was found in patients with high risks of stroke and many comorbidities [48]. Patients with AF using DOACs showed greater persistence than those prescribed with VKAs due to the fixed dose and fewer drug-drug or drug-food interactions [49]. Nevertheless, the medication adherence of anticoagulants would decrease over time [50]. Therefore, patient adherence must be improved to guarantee the effective and safe anticoagulation treatment in NVAF patients with diabetes.

Comedication plays an important role in the effectiveness and safety of anticoagulation management of patients with diabetes and NVAF. Metformin and sulfonylureas, the most widely used glucose-lowering agents, generally increase the risk of bleeding with the concurrent use of warfarin [51]. However, Stage et al. [52] found that initiation of metformin or sulfonylureas could decrease the international normalized ratio (INR) levels among users of vitamin K antagonists, thus leading to a reduced risk of bleeding. Nam et al. [53] reported that use of sulfonylureas or metformin is not associated with an increased rate of serious bleeding in warfarin users. Different from VKAs, DOACs are metabolized via P-glycoprotein (P-gp) transporter and CYP3A4 (rivaroxaban and apixaban) and usually have few drug-drug interactions with commonly used drugs. Nevertheless, antiarrhythmic drugs prescribed for patients with AF are mostly P-gp inhibitors (e.g., verapamil, dronedarone, amiodarone, ranolazine, and quinidine), which may increase the plasma levels of DOACs. Therefore, the avoidance or dose reduction use of DOACs is recommended with concomitant antiarrhythmic drugs (verapamil or dronedarone) by the latest guidance [54]. Statins are also commonly prescribed drugs for patients with diabetes to reduce the risks of cardiovascular events. Statins increase INR in warfarin users [55, 56], leading to high risks of bleeding. Although statins are metabolized via P-gp or CYP3A4, no relevant interaction was found with dabigatran [57], edoxaban [58], or rivaroxaban [59].

Confounding by unmeasured variables is a major problem in real-world cohort studies comparing interventions. Among the included studies, heterogeneity was found in some endpoints of this meta-analysis. Therefore, a sensitivity analysis was performed by deleting one study at a time, and no individual study led to the heterogeneity. The included studies were then carefully examined, and some possible causes for the heterogeneity were identified. First is the diversity in the definition of major outcomes. For example, the definition of major bleeding included intracranial hemorrhage, gastrointestinal bleeding, and other sites of critical bleeding in the studies of Chan et al. [20] and Lip et al. [21], but only intracranial bleeding and gastrointestinal bleeding were considered in the work of Baker et al. [16]. In the study of Hsu et al. [18], haematuria was included in addition to intracranial bleeding and gastrointestinal bleeding; hence, inconsistency may result in heterogeneity. Second, the propensity score matching method to balance residual confounding was used by six studies [16–18, 20–22] but not by one work [19], and this condition may also lead to heterogeneity. Moreover, two of the selected studies [19, 22] had a relatively small sample size and a low number of endpoint events that may limit the statistical power. Lastly, the population was different across these retrospective cohorts: three was comprised of Asians [18–20], three mainly included US patients [16, 17, 21], and one involved Europeans [22]. In summary, all the possible reasons discussed above may lead to the heterogeneity of the included observational studies. However, the use of a random-effect model may help mitigate the effect of heterogeneity on internal validity.

In conclusion, in this meta-analysis of seven observational studies with more than 240,000 patients with NVAF and diabetes, DOACs significantly reduced the risks of stroke, ischemic stroke, SSE, and myocardial infarction and borderline significantly reduced the MACE rate compared with VKAs. Moreover, the risks of major bleeding, intracranial hemorrhage, and major gastrointestinal bleeding were also decreased in patients treated with DOACs. Individual DOAC versus VKAs showed similar or reduced risks of thromboembolic and bleeding events except for edoxaban regarding gastrointestinal bleeding. Further real-world studies in relation to edoxaban are needed. The results supported the advantage of DOACs over VKAs regarding efficacy and safety in patients with NVAF and diabetes in the real world.

### 4.1. Limitations

This study has several limitations. First, given that all the included studies were retrospective observational cohorts, selection bias, misclassification, and residual confounding from unobserved or unmeasured covariates across studies cannot be excluded [60], and thus may affect the internal validity of this work. Second, laboratory data such as international normalized ratio (INR) for patients treated with warfarin were not available. Hence, the proportion of time in therapeutic range (TTR) was indeterminable. An INR of 2.0-3.0 is recommended as the optimal therapeutic range for warfarin users. The poor INR control of warfarin treatment may exaggerate the superiority of DOACs over VKAs in efficacy and safety endpoints. Nevertheless, the pattern of warfarin prescription and management in routine practice allow the study results to accurately reflect real-world situations. Third, diabetic patients usually have poor renal conditions that may affect the use of DOACs in specific patient populations. However, renal function laboratory data were lacking across studies; thus, the residual confounding could not be excluded. Finally, glycated hemoglobin is closely associated with the risk of stroke in AF patients with diabetes [61], but only three studies [18, 19, 22] provided the related data. Therefore, the quality of glycemic control and the impact of glycemic levels on clinical outcomes were not examined in this high-risk diabetic NVAF population.

## 5. Conclusions

Among the patients with NVAF and diabetes in real-world clinical settings, DOACs showed superior efficacy and safety profile over VKAs, and they significantly reduced risks of stroke, ischemic stroke, SSE, myocardial infarction, major bleeding, intracranial hemorrhage, and major gastrointestinal bleeding.

## Figures and Tables

**Table 1:** Stratified analysis of efficacy outcomes according to anticoagulant mechanism.

	Number of reports	Pooled RR (95% CI)	*p* value	*I* ^2^ (%)
*Stroke*				
Overall estimation	4	0.56 (0.45-0.70)	<0.0001	78
Anti-IIa agents	1	0.64 (0.48-0.85)	0.002	
Anti-Xa agents	3	0.54 (0.41-0.71)	<0.0001	85
*Ischemic stroke*				
Overall estimation	6	0.61 (0.48-0.78)	<0.0001	79
Anti-IIa agents	1	0.80 (0.58-1.10)	0.17	
Anti-Xa agents	5	0.58 (0.43-0.77)	<0.0001	82
*Stroke/systemic embolism*				
Overall estimation	8	0.80 (0.68-0.95)	0.01	88
Anti-IIa agents	2	0.79 (0.55-1.13)	0.19	82
Anti-Xa agents	6	0.81 (0.65-1.00)	0.047	90
*Myocardial infarction*				
Overall estimation	8	0.69 (0.55-0.88)	0.002	61
Anti-IIa agents	2	0.66 (0.35-1.25)	0.201	52
Anti-Xa agents	6	0.67 (0.50-0.91)	0.011	68
*MACE*				
Overall estimation	5	0.87 (0.75-1.00)	0.049	82
Anti-IIa agents	1	0.91 (0.81-1.01)	0.084	
Anti-Xa agents	4	0.85 (0.70-1.04)	0.112	87

Anti-IIa agents include dabigatran. Anti-Xa agents include apixaban, edoxaban, and rivaroxaban. CI: confdence interval; MACE: major adverse cardiac events; RR: relative risk.

**Table 2:** Stratified analysis of safety outcomes according to anticoagulant mechanism.

	Number of reports	Pooled RR (95% CI)	*p* value	*I* ^2^ (%)
*Major bleeding*				
Overall estimation	10	0.75 (0.63-0.90)	0.002	94
Anti-IIa agents	2	0.58 (0.52-0.64)	<0.0001	0
Anti-Xa agents	8	0.81 (0.66-0.99)	0.044	95
*Intracranial hemorrhage*				
Overall estimation	13	0.50 (0.44-0.56)	<0.0001	22
Anti-IIa agents	3	0.39 (0.31-0.49)	<0.0001	0
Anti-Xa agents	10	0.52 (0.47-0.59)	<0.0001	0
*Major gastrointestinal bleeding*				
Overall estimation	12	0.77 (0.62-0.95)	0.016	93
Anti-IIa agents	3	0.64 (0.49-0.83)	0.001	66
Anti-Xa agents	9	0.83 (0.64-1.07)	0.15	94

Anti-IIa agents include dabigatran. Anti-Xa agents include apixaban, edoxaban, and rivaroxaban. CI: confidence interval, RR: relative risk.

## Data Availability

The data used to support the findings of this study are included within the supplementary information file.

## Supplementary Materials

Supplementary Materials S1 Table 1: NOS for assessment of quality of included studies. S2. Table 2: baseline characteristics of included studies. S3. Table 3: definitions of safety and efficacy endpoints in the 7 included studies. Figure S1: flowchart diagram illustrating study selection methodology. Figure S2: forest plot comparing DOACs vs. VKAs regarding stroke in real-world NVAF patients with diabetes. Figure S3: forest plot comparing DOACs vs. VKAs regarding ischemic stroke in real-world NVAF patients with diabetes. Figure S4: funnel plots of the reported outcomes. Figure S5: Egger's tests of the reported outcomes.
