# Supplementary material for: Efficacy and Safety of Direct Oral Anticoagulants in Patients with Diabetes and Nonvalvular Atrial Fibrillation: Meta-Analysis of Observational Studies
Source: Cardiovasc Ther. 2021 Oct 11;2021:5520027. doi: 10.1155/2021/5520027 (PMC8523231; doi:10.1155/2021/5520027)
Supplement: Supplementary Materials — S1 Table 1: NOS for assessment of quality of included studies. S2. Table 2: baseline characteristics of included studies. S3. Table 3: definitions of safety and efficacy endpoints in the 7 included studies. Figure S1: flowchart diagram illustrating study selection methodology. Figure S2: forest plot comparing DOACs vs. VKAs regarding stroke in real-world NVAF patients with diabetes. Figure S3: forest plot comparing DOACs vs. VKAs regarding ischemic stroke in real-world NVAF patients with diabetes. Figure S4: funnel plots of the reported outcomes. Figure S5: Egger's tests of the reported outcomes. [file 5520027.f1.doc]

**Supplementary Material**

**Efficacy and Safety of Direct Oral Anticoagulants in Patients with Diabetes and Non-valvular Atrial Fibrillation****: Meta-Analysis of Observational Studies**

Bo Cao^1^, Xingcan Yao^1^, Lifang Zhang^1^, Xiaobo Hu^1^, Min Chen^2^, Mingfeng Shen^1^, Lan Xu^1^

^1^Department of Clinical Pharmacy, Affiliated Ninth Hospital of Suzhou University, Suzhou, China; ^2^Department of Cardiovascular Medicine, Affiliated Ninth Hospital of Suzhou University, Suzhou,China

**S1: Table 1 NOS for assessment of quality of included studies**

| **Included studies** | **Selection (0-4 points)** | | | | **Comparability (0-2 points)** | | **Outcome (0-3 points)** | | | **Total points** |
| --- | --- | --- | --- | --- | --- | --- | --- | --- | --- | --- |
|  | **Representativenes s of**  **Exposed Cohort** | **Selection of Non-Exposed Cohort** | **Ascertainment Of Exposure** | **Demonstration That Outcome of Interest Was Not Present at Start of Study** | **Adjust for the important Risk factors** | **Adjust for other risk factors** | **Assessment of outcome** | **Follow-up length** | **Loss to follow-up rate** |  |
| Baker 2019 | * | * | * |  | * | * | * | * | * | 8 |
| Coleman 2018 | * | * | * |  | * | * | * | * | * | 8 |
| Hsu 2018 | * | * | * |  | * | * | * | * | * | 8 |
| Wang 2020 | * | * | * | * | * | * | * |  |  | 7 |
| Chan 2020 | * | * | * |  | * | * | * | * | * | 8 |
| Lip 2020 | * | * | * |  | * | * | * | * | * | 8 |
| Russo 2020 | * | * | * |  | * | * | * | * | * | 8 |

The Newcastle-Ottawa Scale (NOS) items, with a total score of 9 points, were used to evaluate the quality of the observational studies, which involve the selection of cohorts (0-4 points), the comparability of cohorts (0-2 points), and the assessment of the outcome (0-3 points).

| **Study** | **Study design** | **Period** | **Hypertension(%)** | **Heart failure(%)** | **Coronary artery disease(%)** | **Peripheral artery disease(%)** | **Prior stroke(%)** | **Smoker (%)** | **HbA**1C(%) | **Population DOAC/VKA** | **Age(years)** | **Male(%)** | ChA2DS2-VASc (median) | **Treatment** | **Control** | **Median follow-up(years)** |
| --- | --- | --- | --- | --- | --- | --- | --- | --- | --- | --- | --- | --- | --- | --- | --- | --- |
| Baker 2019 | Retrospective  cohort | 2012.01-2017.12 | 86.5/86.5 | 33.0/33.4 | 5.3/5.1 | 10.7/11.2 | 9.3/9.3 | 6.1/6.1 | NA | 10700/  13946 | 70/70  (median) | 64.7/62.7 | 4.0/4.0 | RIV | WAR | 1.4 |
| Coleman 2018 | Retrospective  cohort | 2011.11-2016.12 | 85.6/85.9 | 24.7/25.7 | NA | 20.3/21.1 | 7.4/7.4 | 5.7/5.5 | NA | 5517/5517 | 70/70  (median) | 63.3/63.5 | 3.0/3.0 | RIV | WAR | 1.5 |
| Hsu 2018 | Retrospective  cohort | 1999.01-2015.12 | 92.0/92.6 | 40.8/39.9 | 66.3/64.0 | 69.9/70.8 | 34.7/34.6 | NA | 7.55/7.47 | 605/606 | 75.1/74.1 | 50.6/50.8 | 6.0/6.0 | RIV;  DAB | WAR | 1.7 |
| Wang 2020 | Retrospective  cohort | NA | 29.0/36.0 | NA | NA | NA | NA | 43.0/  44.0 | 7.55/7.21 | 201/383 | 69.5/68.7 | 59.0/55.0 | NA | RIV | WAR | 1.5 |
| Chan 2020 | Retrospective  cohort | 2012.06-2017.12 | 80.1/79.6 | 13.2/13.8 | NA | 9.6/9.5 | 21.8/21.1 | NA | NA | 20967/  5812 | 74.6/74.5 | 53.7/53.5 | 4.5/4.4  (mean) | API;  DAB;  EDO;  RIV | WAR | 2.75 |
| Lip 2020 | Retrospective  cohort | 2013.01-2015.09 | 94.8/94.8 | 36.7/37.0 | 55.0/54.7 | 25.8/26.3 | 13.9/13.9 | NA | NA | 92635/  92635 | 75.2/75.3 | 55.0/54.9 | 4.0/4.0 | API;  DAB;  RIV | WAR | 0.58 |
| Russo 2020 | Retrospective  cohort | 2013.03-2018.07 | 53.1/55.6 | 20.8/21.4 | NA | NA | 28.8/29.4 | NA | 8.2/8.9 | 135/135 | 69.2/70.3 | 58.6/59.3 | 4.3/4.5  (mean) | EDO | VKA | 2.25 |

**S2: Table 2 Baseline characteristics of included studies**

CHA2DS2-VASc score: congestive heart failure, 1 point; hypertension, 1 point; age ≥ 75 years, 2 points; diabetes mellitus, 1 point; previous stroke, transient ischemic attack or thromboembolism, 2 points; vascular disease, 1 point; age 65–74 years, 1 point; female sex, 1 point

DOAC=direct antagonist oral anticoagulants; VKA=vitamin K antagonists; WAR=warfarin; DAB=dabigatran; RIV=rivaroxaban; API=apixaban; EDO=edoxaban; NA=not available.

**S3: Table 3** **Definitions of safety and efficacy endpoints in the 7 included studies**

| **Study** | **Stroke** | **Ischemic stroke** | **Stroke or systemic embolism** | **Myocardial infarction** | **Major adverse cardiac events** | **Major bleeding** | **Intracranial**  **hemorrhage** | **Major gastrointestinal bleeding** |
| --- | --- | --- | --- | --- | --- | --- | --- | --- |
| Baker 2019 | NA | ischaemic stroke | NA | myocardial infarction | ischaemic stroke or MI | intracranial or gastrointestinal | intracranial bleeding | gastrointestinal bleeding |
| Coleman 2018 | ischaemic stroke (ICD-10=I63; I64.9), haemorrhagic stroke  (ICD-10=I60-I62) | ischaemic stroke (ICD-10=I63; I64.9) | ischaemic stroke (ICD-10=I63; I64.9), haemorrhagic stroke  (ICD-10=I60-I62) or systemic embolism (ICD-10=I74) | NA | NA | major bleeding | intracranial  hemorrhage | gastrointestinal bleeding |
| Hsu 2018 | NA | Stroke (infarct)  ICD9:  43301,43311,43321,43331,43381,43391,43401,43411,43491 | NA | myocardial infarction | NA | intracranial  hemorrhage; gastrointestinal haemorrhage; haematuria | intracranial  bleeding (ICD-9 430–432) | ICD:9  53021,5307, 53082,53100,53101,53120,  53121,53140,53141,53160,53161,53200,  53201,53220,53221,53240,53241,53260,  53261,53300,53301,53320,53321,53340,  53341,53360,53361,53400,53401,53420,  53421,53440,53441,53460,53461,53501,  53511,53521,53531,53541,53551,53561,  53571,53783,56202,56203,56212,56213,  5693, 56985,56986,5780, 5781, 5789 |
| Wang 2020 | NA | ischaemic stroke | NA | myocardial infarction | NA | NA | intracranial  bleeding | gastrointestinal bleeding |
| Chan 2020 | NA | ICD-9  433, 434, 436;  ICD-10  I63, I64 | ischemic stroke/systemic embolism | ICD-9  410;  ICD-10: I21-I23 | ischemic stroke/systemic embolism;  acute myocardial infarction | intracranial hemorrhage; gastrointestinal bleeding; other critical site bleeding | ICD-9  430, 431, 432, 852, 853;  ICD-10  I60, I61, I62 | ICD-9  456.0, 456.2, 455.2, 455.5, 455.8, 530.7, 530.82, 531.0-531.6, 532.0-532.6, 533.0-533.6, 534.0-534.6, 535.0-535.6 537.83, 562.02, 562.03, 562.12 562.13 568.81, 569.3, 569.85, 578.0, 578.1, 578.9  ICD-10  K22.6, K25.0, K25.2, K25.4, K25.6, K26.0, K26.2, K26.4, K26.6, K27.0, K27.2, K27.4, K27.6, K28.0, K28.2, K28.4, K28.6, K29.0, K62.5, K92.0, K92.1, K92.2 |
| Lip 2020 | ICD-9-CM:  haemorrhagic stroke430.xx-432.xx  ischaemic stroke 433.x1, 434.x1, 436 | ICD-9-CM:  ischaemic stroke 433.x1, 434.x1, 436 | ICD-9-CM:  haemorrhagic stroke430.xx-432.xx  ischaemic stroke 433.x1, 434.x1, 436  systemic embolism  444.x, 445.x | NA | NA | major intracranial hemorrhage; major gastrointestinal bleeding; major other hemorrhage | ICD-9-CM:  430, 431, 432.0, 432.1, 432.9, 852.0x, 852.2x, 852.4x, 853.0x, | ICD-9-CM:  456.0, 456.20, 530.82, 531.0x, 531.2x, 531.4x, 531.6x, 532.0x, 532.2x, 532.4x, 532.6x, 533.0x, 533.2x, 533.4x, 533.6x, 534.0x, 534.2x, 534.4x, 534.6x, 535.01, 535.11, 535.21, 535.31, 535.41, 535.51, 535.61, 537.83, 562.02, 562.03, 562.12, 562.13, 568.81, 569.3, 569.85, 578.x |
| Russo 2020 | NA | ischaemic stroke | systemic embolism | NA | NA | ISTH | intracranial hemorrhage | NA |

Abbreviations: MI: myocardial infarction; ICD 9-CM: International Classification of Disease 9^th^ Clinical Modification; ICD 10-CM: International Classification of Disease 10^th^ Clinical Modification; ISTH, the International Society on Thrombosis and Haemostasis. NA, not available

**
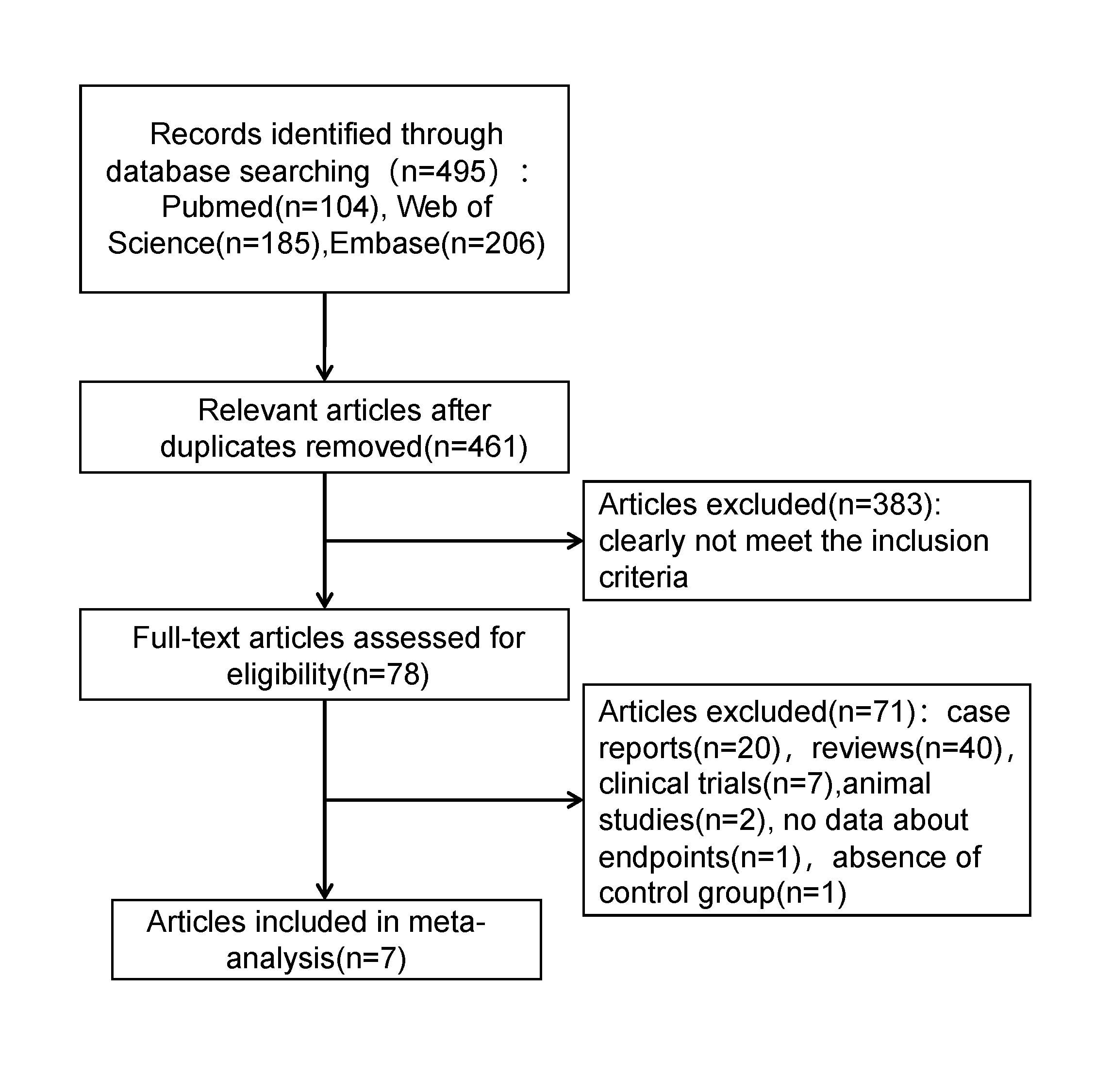
**

**Fig S1** Flowchart diagram illustrating study selection methodology.


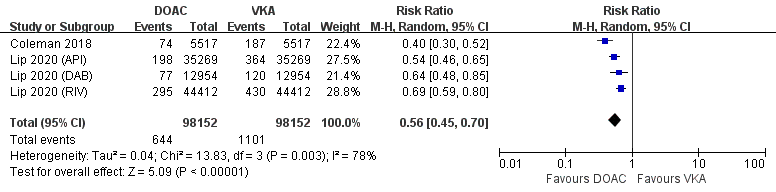


**Fig S2** Forest plot comparing DOACs vs VKAs regarding stroke in real-world NVAF patients with diabetes. NVAF, non-valvular atrial fibrillation; DOACs, direct oral anticoagulants; VKAs, vitamin K antagonists.


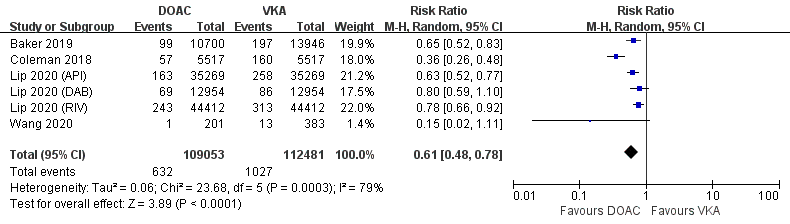


**Fig S3** Forest plot comparing DOACs vs VKAs regarding ischemic stroke in real-world NVAF patients with diabetes. NVAF, non-valvular atrial fibrillation; DOACs, direct oral anticoagulants; VKAs, vitamin K antagonists.

**
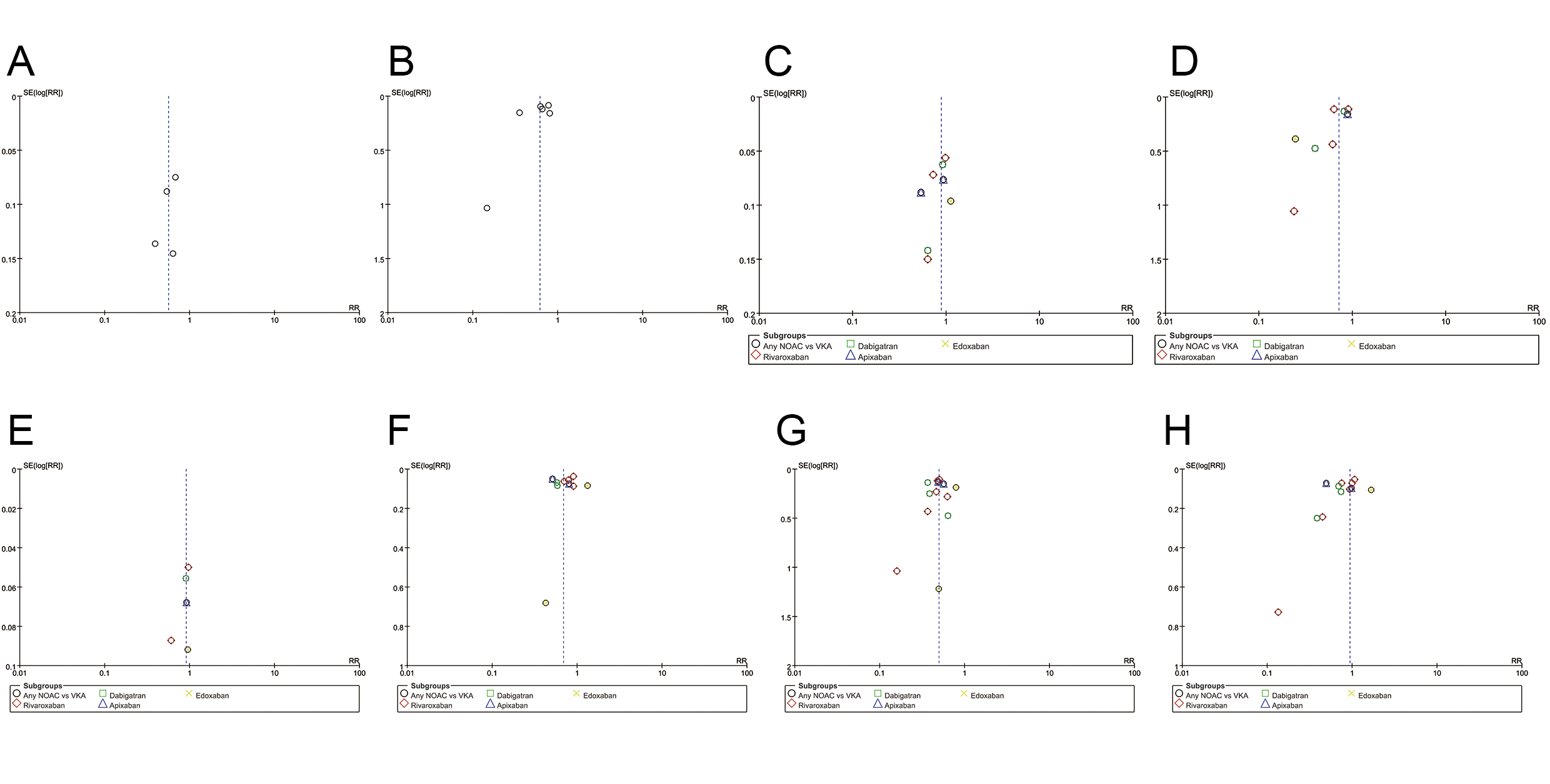
**

**Fig S4** Funnel plots of the reported outcomes. (A) stroke; (B) ischemic stroke; (C)stroke or systemic embolism; (D) myocardial infarction; (E) MACE; (F) major bleeding; (G) intracranial bleeding; and (H) major gastrointestinal bleeding. MACE, major adverse cardiac events; RR=risk ratio; SE=standard error.


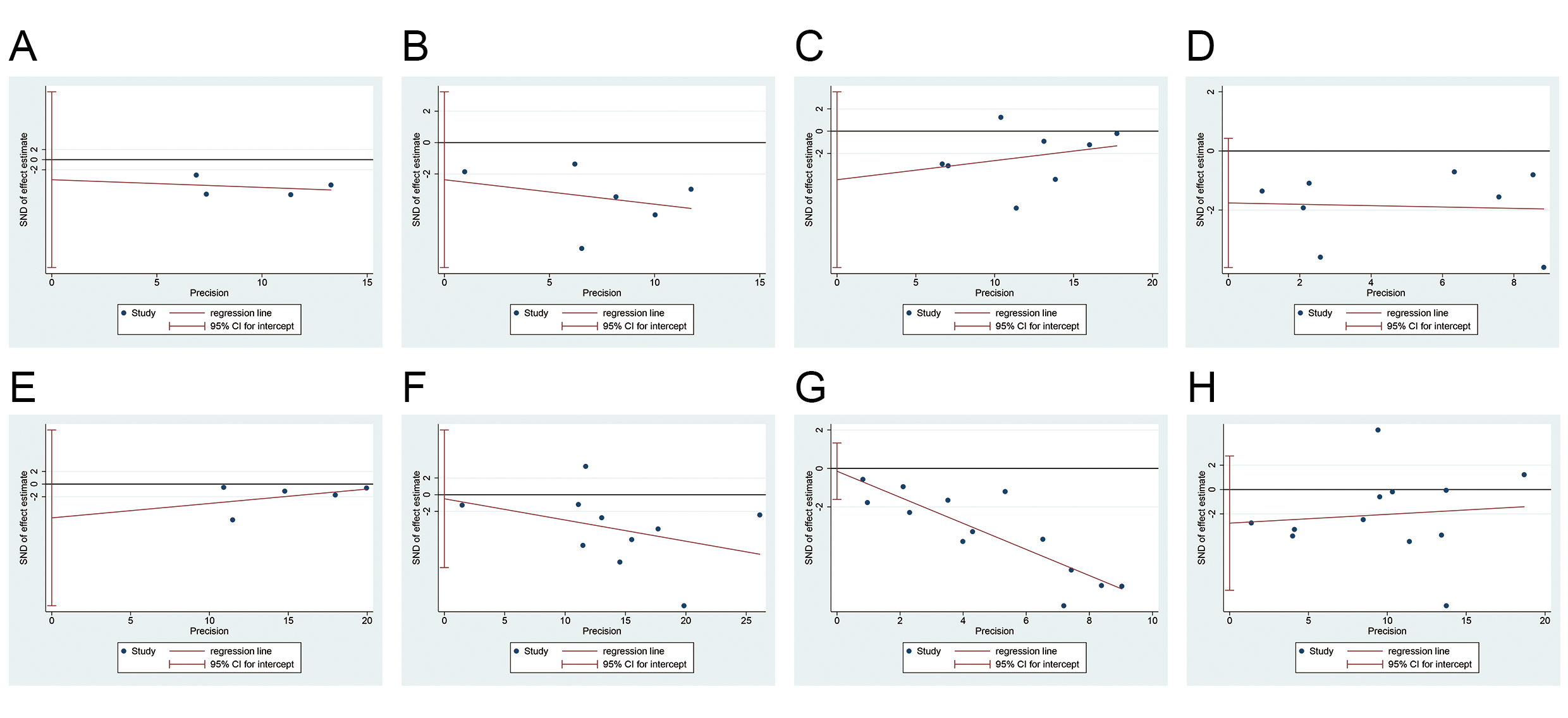


**Fig S5** Egger’s tests of the reported outcomes: (A) stroke (p=0.429); (B) ischemic stroke (p=0.306); (C)stroke or systemic embolism (p=0.225); (D) myocardial infarction (p=0.097); (E) MACE (p=0.308); (F) major bleeding (p=0.894); (G) intracranial bleeding (p=0.833); and (H) major gastrointestinal bleeding (p=0.291). MACE, major adverse cardiac events; CI: confidence interval.
